# The effects of a nutrient supplementation intervention in Ghana on parents’ investments in their children

**DOI:** 10.1371/journal.pone.0212178

**Published:** 2019-03-13

**Authors:** Katherine P. Adams, Seth Adu-Afarwuah, Helena Bentil, Brietta M. Oaks, Rebecca R. Young, Stephen A. Vosti, Kathryn G. Dewey

**Affiliations:** 1 Program in International and Community Nutrition, Department of Nutrition, University of California, Davis, Davis, CA, United States of America; 2 Department of Nutrition and Food Science, University of Ghana, Legon, Accra, Ghana; 3 Department of Nutrition and Food Sciences, University of Rhode Island, Kingston, RI, United States of America; 4 Department of Agricultural and Resource Economics, University of California, Davis, Davis, CA, United States of America; Kansas State University, UNITED STATES

## Abstract

A child’s endowment is a reflection of his/her genetic makeup and the conditions faced in early life. Parents build on their child’s endowment by investing resources in their child, and together, a child’s endowment and subsequent investments act as input into important later-life outcomes. A positive or negative shock to a child’s endowment can have a direct biological effect on a child’s long-term outcomes but may also affect parents’ decisions about investments in the health and human capital of their children. Using follow-up data collected several years after a randomized trial in Ghana, we explored whether maternal and child supplementation with small-quantity lipid-based nutrient supplements (SQ-LNS) throughout much of the first 1,000 days influenced parental investments in the health and human capital of their children. Across the domains of family planning, breastfeeding, health, education, and paternal financial support, we found that, in general, the intervention did not affect investments in the treated child nor his/her untreated siblings. These results suggest that given production technologies, constraints, and preferences, the intervention either did not change parents’ optimal investment strategies or that the effects of the intervention, namely increased birth size and attained length at 18 months of age, were too small for parents to perceive or to have any meaningful impact on parents’ expectations about the returns to investments in their children.

## Introduction

The foundation of a child’s growth, health, and development is established during the first 1,000 days, from conception to 24 months of age [1]. This foundation, or early life “endowment”, is a reflection of a child’s genetic makeup and the conditions faced in early life. Parents build on their child’s endowment by investing resources in their child, examples of which include spending time engaging in developmentally stimulating activities with their child, nourishing their child, maintaining their child’s health, and sending their child to school. Together, a child’s endowment and subsequent investments in the child act as input into important later-life outcomes, including health, cognitive ability, and economic productivity in adulthood.

The influence of a child’s endowment, particularly a child’s nutritional status, on these later-life outcomes is well-established [1–3] and recognition of this has contributed to the elevation of maternal and early childhood nutrition as a top global health and economic development priority [4–6]. Along with our understanding of the long-term implications of nutrition in early life, work elucidating the dynamic nature of human capital formation and the key role of timely parental investments [7–9] has inspired and informed theoretical and empirical interest in the relationship between a child’s early life endowment and subsequent parental investments in the health and human capital of their children (see Almond and Mazumder [10] and Almond, Currie [11] for an overview of this literature). That is, there is broad interest in whether, and in what direction, parents respond to a positive or negative shock to a child’s endowment by altering investments in the child.

When parental investments move in the same direction as a shock to their child’s endowment, the investments are said to be reinforcing (that is, parents respond to a positive shock by increasing investments), while parental investments that move in the opposite direction of a shock are termed compensatory (a positive shock reduces investments). Theoretically, the direction of the parental investment response is ambiguous *a priori*, as it depends on the nature of the production technologies transforming a child’s endowment and parental investments into the child’s health and human capital as well as a number of other factors [10, 11]. The degree of substitutability between a child’s endowment and subsequent investments as inputs into the production of health and human capital, for example, would influence whether and in what direction parental investments respond to shocks. Likewise, dynamic complementarities between a child’s endowment and investments such that the future productivity of investments depends on the endowment would also influence the response. These abstract concepts, reflected on a basic level in parents’ beliefs about how well investments in their child will perform, along with parental preferences (e.g., do parents seek equity among their children or to maximize the total productivity of their investments in their children?), household resource constraints, parental perceptions or knowledge about their children’s endowments, and parents’ knowledge of and expectations about the returns to early-life investments would all play a role in determining the nature of the response. If parental investments respond to the strength or quality of their children’s endowments, then the full effect of a shock to a child’s endowment on the child’s long-term outcomes will be determined in part by the degree to which changes in parental investments either strengthen or diminish the purely biological effects of the shock. From a policy perspective, then, understanding if and how parental investments respond to interventions aimed at improving a child’s endowment may provide insight into whether additional efforts, such as providing information and/or incentives to parents, might be necessary to help ensure that parents do not reduce critical investments in their children and, ultimately, that the full benefits of the intervention on the child’s long-term outcomes are realized.

In this study, we explored whether maternal and child supplementation with small-quantity lipid-based nutrient supplements (SQ-LNS) in Ghana throughout much of the first 1,000 days influenced concurrent and subsequent parental investments in their children. Lipid-based nutrient supplements (LNS) deliver micronutrients embedded in a food base, thereby also providing energy, protein, and essential fatty acids [12]. The “small quantity” version of LNS, which provide ~110 kcal per 20 gram daily serving, were designed to prevent undernutrition during the first 1,000 days. In a randomized controlled trial of SQ-LNS in Ghana, the provision of SQ-LNS to mothers during pregnancy increased birth size, particularly among first-time mothers [13], and maternal supplementation during pregnancy and the first six months postpartum plus infant supplementation from 6–18 months of age increased mean attained length at 18 months of age [14]. Using follow-up data collected several years after the completion of the randomized trial in Ghana, we explored whether the intervention influenced parents’ investments in their child’s health and human capital.

An intervention that affects the endowment of one child in a family but not others may change the relative marginal returns to investing across children within a household. Given this, and because parents with multiple children have to decide how scarce resources are allocated among their children, we also assessed whether the intervention affected investments in the older and younger siblings of the child who participated in the trial. Across five domains of parental investments (family planning, breastfeeding, health, education, and paternal financial support), we found very limited evidence to support the hypothesis that the intervention induced parents to change how they invested in either the treated child or in their other children.

The bulk of the evidence on early childhood endowment shocks and parental investments is from developed countries, but tighter resource constraints, differences in access to schooling and employment opportunities, as well as different social contexts mean that the investment strategies of parents in low- and middle-income countries (LMICs) might differ from those of parents in higher-income countries. The evidence from LMIC settings is mixed with regard to whether and how parental investment decisions are shaped by their children’s endowments. Adhvaryu and Nyshadham [15] found evidence of reinforcing investments: children who were exposed to a prenatal iodine supplementation program in Tanzania were breastfed longer and were more likely to be vaccinated than unexposed children. Because prenatal iodine deficiency is linked to impaired neurological development, the authors concluded that parents reinforced improvements in their child’s cognitive endowment by increasing investments in their child’s health. The authors also found evidence that parental investments in the exposed child’s siblings were affected: older siblings were more likely to be vaccinated. Likewise, Venkataramani [16], who found positive effects of early life malaria eradication in Mexico on adult cognition, also found that the parents of children who benefitted from malaria eradication efforts during their birth year chose to enroll those children in school at an earlier age than parents of children who did not have early exposure to the eradication efforts.

Other studies have found compensating or mixed parental investments. For example, Bharadwaj, Eberhard [17] analyzed panel data from Chile and found a negative correlation between birth weight and parental investments in education, implying that parents of lower birth weight children were acting to compensate for a lower endowment by devoting relatively more resources to the child’s education. In another study, Miller [18] found some evidence that parents of children exposed to prenatal seasonal food scarcity in Ethiopia compensated for this relatively mild nutritional shock through investing more in the child’s nutrition and education, though these compensatory investments were limited and did not manifest until children were older (around 12 years of age). Leight [19] explored intrahousehold variation in parental investments in siblings in rural China and, using an instrumental variables strategy, found that expenditures on education (though not health) were higher for the relatively less endowed sibling as measured by height-for-age. Yi, Heckman [20] exploited an extensive dataset on Chinese twins to explore parental investment responses to a health insult suffered in early childhood. The authors found that when one twin experienced a serious disease between birth and age three, which was assumed to vary exogenously between twins, parents spent more on the health but less on the education of the twin that suffered the negative shock compared to the other twin, suggesting that the nature of parental investments responses may differ across different domains of human capital. Similarly, Ayalew [21] found that parents in Ethiopia compensated for differences in siblings’ health endowments by investing more in the health of the less endowed sibling but adopted a reinforcing strategy with respect to educational investments.

Finally, some studies have found that parents adopt a neutral investment strategy that does not change in response to a child’s endowment. For example, using birth records data from infants in Chile, Bharadwaj, Løken [22] found that being classified as very low birthweight (<1500g), which resulted in extra medical attention for the infant at birth, had no effect on subsequent parental investments in the child in terms of school quality or parental time spent with the child. Abufhele, Behrman [23] likewise found no difference in parental investments in the health or education of Chilean twin pairs with differing endowments as measured by birth weight.

Together, the mixed results in the literature from LMIC contexts suggest that parental investment responses to early life endowments may be context-specific and depend on the dimension of a child’s endowment being considered (e.g., cognitive capacity vs physical size) and/or the domain of the investment response (e.g., investments in health vs education). This study contributes new evidence based on the randomized provision of a nutrient supplement to mothers and their infants that had a modest but statistically significant effect on measures of a child’s early life endowment, namely birth size and attained length at 18 months of age. We assessed the effect of the intervention on a range of investments, some occurring during the intervention and others several years later, in the treated child in the household. We also assessed the effect of the intervention on parental investments in their untreated children. As such, this analysis offers insight into several dimensions of the possible effects of an early-life nutrition intervention on parents’ investments in their children.

## Methods

### Randomized trial and follow-up

The randomized controlled trial, which is described elsewhere in detail [13, 14], was designed to test the efficacy of SQ-LNS provided to mothers during pregnancy and the first six months postpartum and then to their infants (referred to as index children throughout) from 6–18 months of age. The trial was conducted between December, 2009 and March, 2014 in several semi-urban communities located along a commercial corridor in the Yilo Krobo and Lower Manya Krobo districts of Ghana. Women were recruited on a rolling basis from late 2009 to late 2011 during routine prenatal visits at the four main health facilities in the area. Women were screened for eligibility, and eligible women who provided informed consent were then enrolled into the trial and individually randomized into one of three intervention groups. One group received daily iron-folic acid capsules throughout pregnancy, a component of the current standard of prenatal care in Ghana, and a daily low-dose calcium placebo capsule for the first six months postpartum (IFA group hereafter). A second group received daily multiple micronutrient capsules during pregnancy and through the first six months postpartum (MMN group hereafter). A third group received 20 grams/day of SQ-LNS formulated for maternal consumption during pregnancy and the first six months postpartum, and their children received 20 grams/day of SQ-LNS formulated for child consumption from 6–18 months of age (LNS group hereafter). Mothers in the IFA and MMN groups were blinded to which of the (identical) capsules they received, and the children of women randomized into either of the capsule groups did not receive any supplementation. The nutrient content of each of the capsules and of the SQ-LNS product for pregnant and lactating women and the SQ-LNS product for children are available in S1 Table.

At enrollment, women were given instructions for consuming their assigned supplement along with a brief nutrition message (which was repeated at 36 weeks of gestation) advising them to continue to eat meat, fish, eggs, fruits, and vegetables whenever possible. Throughout pregnancy, the study staff made biweekly home visits to deliver supplements to the women and to collect data on morbidity and supplement use. After birth, women and their infants were visited weekly. When the index children reached 6 months of age, women in the LNS group received instruction for feeding the index child SQ-LNS, and women in all groups received a nutrition message about the importance of continued breastfeeding and feeding their children a variety of nutritious foods (which was repeated at 12 months of age). Throughout the trial, the information conveyed to study participants and scheduled contact between participants and study staff were, by design, uniform across intervention groups. The texts of the instructional and nutrition messages are available in S1 Methods.

The primary outcomes of the original trial were birth size and attained length of the index child at 18 months of age. Infant development and other secondary outcomes were also assessed. Mean birth weight was higher among newborns of mothers in the LNS group compared to the IFA group, while mean newborn length and length-for-age z-scores (LAZ) did not differ by intervention group. [13]. Several factors modified the effect of LNS on birth outcomes, with maternal parity being a particularly strong and consistent source of heterogeneity in observed effects. Among first time mothers, newborns in the LNS group had higher mean birth length, weight, and head circumference compared to newborns in the IFA group and compared to newborns in the MMN group, whereas there were no significant differences in birth outcomes among multiparous mothers. At 18 months of age, differences in index children’s attained size reflected differences at birth. Mean weight, weight-for-age z-score, length, and length-for-age z-score were higher in the LNS group compared to the IFA group and compared to the MMN group [14]. In terms of development, while infants in the LNS group were more likely to be walking by 12 months of age than infants who did not receive SQ-LNS, there were no differences in motor, language, socio-emotional, or executive function between groups at 18 months of age [24].

Between January, 2016 and December, 2016 when the index children were between 4 and 6 years of age, we re-contacted households from the original study to collect data on growth, development, and other outcomes. As a component of the follow-up data collection activities, we collected data on parental investments in the index child and the index child’s siblings. The main trial was registered at clinicaltrials.gov as NCT00970866, and the follow-up study was approved by the ethics committees of the University of California, Davis, the Ghana Health Service, and the University of Ghana College of Basic and Applied Sciences. Written informed consent was obtained from all mothers and caregivers prior to their participation in the follow-up study.

### Empirical methods

As previously described, mothers were enrolled in the original trial on a rolling basis over a two year period, so during the follow-up data collection period, the index children ranged in age from approximately 4 to 6 years. During follow-up, we collected data on parental investments in the index child as well as his/her close siblings, where close siblings included all younger biological siblings and older biological siblings who were under 10 years of age on January 1, 2016. In most cases, the survey respondent was the index child’s mother, but in cases where the index child no longer resided with his/her biological mother (~7% of index children) as a result of maternal death, divorce, relocation, etc., the survey respondent was the child’s primary caregiver. The factors that influenced parental decision making with respect to investments in both the index child and his/her siblings were likely to be different in circumstances in which the index child no longer lived with his/her mother, and respondents other than the index child’s mother were unlikely to provide accurate information on e.g., the duration of breastfeeding or timing of the introduction of complementary foods. As such, when the index child no longer resided with his/her biological mother, we collected data only on a subset of investments in the index child (education and paternal financial support) and did not collect data on investments in the index child’s siblings.

Given the age range of the index children, and to ensure that analyses of investments in siblings were comparable across index children, we limited the analysis of investments in younger siblings to the index child’s nearest younger sibling born within four years of the index child. The analysis was limited to the nearest younger sibling because only a very small number of household (<2%) had more than one younger sibling born within four years of the index child. All older siblings under age 10 were included in the analysis. Investments in the index children and their siblings were analyzed separately by cohorts of children: (1) index children, (2) the index children’s next closest younger siblings and (3) the index children’s older siblings under age 10.

#### Outcome variables

Outcome variables, or parental investments, were grouped into five domains: family planning, breastfeeding, health, education, and paternal financial support. Each of these outcome variables is a parental investment insofar as each of them represents a choice some parents made to allocate scarce resources (energy, time, money, etc.) toward their child’s health and human capital that could have been allocated elsewhere.

The sole outcome in the family planning domain was an ordered categorical variable describing birth spacing after the index child. The categories reflected whether the index child had no living biological siblings born within 48 months of him/her, had a living younger sibling that was born between 24–48 months of him/her, or had a living younger sibling that was born within 24 months of him/her. These categories were chosen to align with the World Health Organization (WHO) recommendation of an interval of at least 24 months between the birth of a live infant and a subsequent pregnancy in order to reduce the risk of adverse maternal and infant health outcomes [25]. With respect to an older sibling (in this case the index child), the time and financial constraints that parents face are tightened when a newborn joins the household. Longer birth intervals can therefore be interpreted as an investment in the index child insomuch as, all else constant, parents have more time to spend with the index child and more money to allocate to the child’s wellbeing until the next child is born. Possibly reflecting these differential constraints, shorter birth intervals have also been shown to be negatively associated with older siblings’ progression in school in sub-Saharan Africa [26] and reading and math scores in the United States [27].

To promote optimal growth, health, and development, the WHO recommends that infants be exclusively breastfed until six months of age, and then complementary foods should be introduced at six months with continued breastfeeding until at least two years of age [28]. These recommendations, which are adopted by the Ghana Health Service, are incorporated into messaging communicated to mothers in Ghana [29] and were also part of the nutrition messaging conveyed to mothers who participated in the randomized trial. In the breastfeeding domain, two parental investments were analyzed to capture adherence to these recommendations. The first was a dichotomous variable reflecting, by maternal recall at the time of the follow-up study, whether the child received his/her first complementary food or drink at 6 months of age versus earlier or later than 6 months. This outcome was analyzed for index children and for younger siblings who were at least six months of age at the time of follow-up data collection. Duration of breastfeeding, which was analyzed for index children only and was again reported by mothers retrospectively, was calculated as a count variable equal to the number of months the child was breastfed before completely weaning. We did not analyze whether or not the child was breastfed (at all) because breastfeeding was nearly universal in the sample population.

The health domain was comprised of four outcomes. The first outcome, ‘child delivered in a health facility’, was analyzed for the younger sibling of the index child only, and was defined as a dichotomous variable indicating whether or not the sibling was delivered in a formal health facility (hospital, clinic, health center, etc.). This outcome was not analyzed for index children because prior to delivery during the intervention, mothers were repeatedly encouraged by the study to deliver at the hospital to facilitate the timely collection of birth outcomes. Further, at the time of the intervention, the National Health Insurance Scheme covered all costs associated with delivery in a formal health facility, and the iLiNS study paid for health insurance for mothers who did not have coverage. The outcome ‘child has health insurance’, which was analyzed for all cohorts, was a dichotomous variable reflecting whether or not the mother indicated that the child was covered by Ghana’s National Health Insurance Scheme. At the time of follow-up, health insurance coverage under the national scheme required that a small fee be paid at initial registration and at each annual renewal of coverage. The health investment ‘mother has child’s health record’ was an indicator of whether or not the mother was able to present the child’s health record, which documented information on the child’s date of birth, immunizations, etc., to the enumerator who conducted the investments interview. This outcome, which was analyzed for all cohorts, was an indicator of whether or not the mother was mindful enough of the child’s health to keep track of the record. And finally in the health domain, mosquito bed net use, analyzed for all cohorts, was captured by the ordered categorical variable ‘bed net use the previous night’ with categories indicating, by maternal report, that the child did not sleep under a bed net the night before interview, that the child slept under a bed net but it was not treated with insecticide, or that the child slept under an insecticide-treated bed net.

In the education domain, the outcome ‘age-appropriate schooling progression’, which was analyzed for index children, was a dichotomous variable reflecting whether or not the child had completed at least an age-appropriate number of terms of schooling at the time of the investment survey. This variable was constructed based on the assumption that if a child turned 4 years old on or before September 1 of a particular academic year, the child should have begun kindergarten (KG1) that academic year. Given that the index children were between 4 and 6 years of age during follow-up, this variable was more a measure of whether the child’s parents had enrolled him/her in school on time rather than an indicator of whether or not the child was doing well enough in school to advance normally from one grade to the next. For the index children’s older siblings, progression through schooling was measured as a count of completed terms of school. For both index children and older siblings, we also assessed the effect of the intervention on whether or not the child was enrolled in a private school, which are common in the study area but have higher costs than the government-run public schools.

Finally, for index children, the single outcome in the paternal financial support domain was an ordered categorical variable indicating the frequency with which the index child’s father, as reported by the child’s mother or primary caregiver, provided financial support to help pay for food, clothes, school fees, etc. for the index child (categories were never, sometimes, often, and always).

#### Estimation

The individually randomized design of the original trial provided exogenous variation in maternal and child supplementation with SQ-LNS. We used this variation to identify the effect of the intervention on each of these parental investments in their children. Combining the IFA and MMN groups (non-LNS group), the effect of receiving SQ-LNS for maternal and child consumption on parental investments in the index child was estimated by regressing the investment outcome of interest on an indicator variable equal to one if the mother and her index child were randomized to receive SQ-LNS and zero otherwise, and a vector of child and household characteristics (described below). Parallel regression models were separately estimated for investments in the index child’s older siblings and the index child’s closest younger sibling.

Dichotomous outcomes were analyzed using probit models. Ordered categorical variables were estimated with ordered probit models. Count variables were estimated using Poisson models, and the count variable ‘completed terms of education’ was analyzed using a negative binomial model with an exposure term set to the maximum possible terms of education, which varied based on the child’s age at the time the investments data were collected. For all analyses of the older siblings’ cohort, standard errors were clustered at the household level.

All regression models were estimated with controls for the age of the index child (as well as the age of the sibling for all sibling analyses) plus an additional set of pre-specified baseline covariates. These pre-specified covariates were sex of the index child, maternal parity at the birth of the index child, maternal height, maternal age, maternal years of education, gender of the head of household, and whether the household had electricity. Among these, specific covariates were included if they were associated with the outcome variable at the 10% level of significance in a bivariate analysis or if they were significantly different in the follow-up sample compared to the sample that was lost to follow-up.

Heterogeneity in the effect of the intervention on each investment outcome was assessed using interaction terms for each of the pre-specified baseline covariates (sex of the index child, maternal parity at the birth of the index child, maternal height, maternal age, maternal years of education, gender of the head of household, and whether the household had electricity) in addition to age and sex of the sibling for analyses of investments in the index child’s older and younger siblings. Statistically significant interactions (p < .10) were further examined using Stata’s ‘margins’ command. For dichotomous effect modifiers, the effect of the intervention by effect modifier was estimated. For continuous effect modifiers, the nature of the heterogeneity was explored at key values along the range of the effect modifier.

All analyses were performed using Stata statistical software (version 15; Stata Corporation, College Station, TX, USA). P-values < .05 were considered statistically significant, while p-values ≥ .05 and < .1 were considered marginally significant.

#### Sensitivity analysis

We performed two sets of sensitivity analyses. First, we estimated all models comparing the three original intervention groups (IFA, MMN, and LNS) rather than the combined IFA and MMN groups compared to the LNS group. Where the null of no difference between the three groups was rejected, p-values for post-hoc pairwise group comparisons were adjusted for multiple comparisons using Sidak’s method.

Also, as described below, there was a relatively high rate of attrition at follow-up, and there were some statistically significant differences in the characteristics of those who were lost to follow-up compared to those who remained in the sample, which could introduce attrition bias. Therefore, as a second sensitivity analysis, we used the inverse probability weighting method [30] to assess the sensitivity of our results to adjusting for potentially non-random attrition. In particular, we calculated inverse probability weights from logit estimates of the probability of remaining in the sample at follow-up. We then reran each regression weighted by the inverse probability of remaining in the sample at follow-up.

## Results

### Attrition and balance

**Fig 1:**
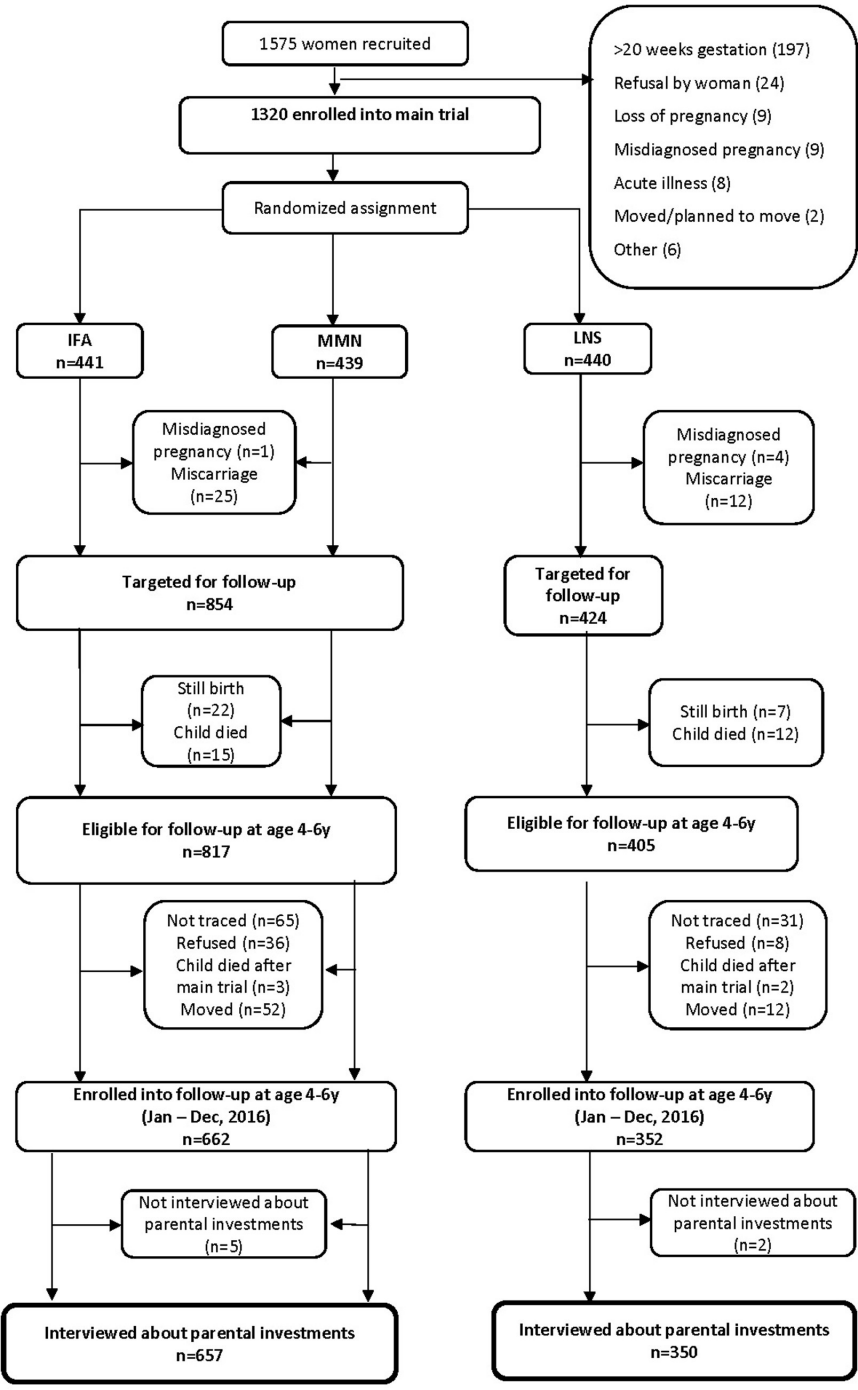
Study profile. IFA = iron-folic acid; MMN = multiple micronutrients; LNS = lipid-based nutrient supplements.

There were 1,320 women originally enrolled in the randomized trial. Excluding misdiagnosed pregnancies (n = 5), miscarriages (n = 37), stillbirths (n = 29), and child deaths before 18 months (n = 27), we targeted the mothers and primary caregivers of 1,222 index children for follow-up data collection on investments in the index child and his/her close siblings. We successfully interviewed 1,007 mothers and primary caregivers (rate of successful follow-up of 82.4%). Based on the full original sample of 1,320 mothers, the overall attrition rate was 23.7%. This rate was significantly higher in the non-LNS group compared to the LNS group (25.3% vs 20.5%, p = 0.049). See Fig 1 for the study profile, from the main trial through the follow-up study.

There were a number of statistically significant differences in the characteristics of the follow-up and lost to follow-up samples (Table 1). At the beginning of follow-up data collection, index children in the follow-up sample were, on average, slightly younger than children who were lost to follow-up (p = 0.013). Mothers in the follow-up sample had had slightly more pregnancies prior to the index child (p = 0.024) and were taller, on average, than mothers who were lost to follow-up (p = 0.040). Households in the follow-up sample were less likely to be female-headed (p = 0.018) and more likely to have electricity than households that were lost to follow-up (p = 0.080. In terms of key outcomes from the original randomized trial, these samples did not differ in mean birth weight of the index children or mean length-for-age z-score of the index children at 18 months of age. As previously noted, in all regression models we controlled for the background characteristics that differed between the samples.

**Table 1:** Characteristics of follow-up and lost to follow-up samples.

Variable	Follow-up Sample		Lost to Follow-Up Sample		P-value
	N	Mean ± SD or %^1^	N	Mean ± SD or %^1^	
Index child age at start of follow-up data collection (y)	1007	4.6 ± 0.6	240	4.7 ± 0.6	0.013^2^
Index child male (%)	1007	48.3	241	53.1	0.176^3^
Maternal parity at birth of index child (n)	1007	2.3 ± 1.3	313	2.1 ± 1.2	0.024^4^
Maternal age* (y)	1007	26.8 ± 5.4	313	26.4 ± 5.8	0.328^2^
Maternal education* (y)	1004	7. 4 ± 3.6	295	7.5 ± 4.0	0.799^2^
Maternal height* (m)	1005	1.59 ± 0.06	311	1.58 ± 0.06	0.040^2^
Head of household female* (%)	1002	26.0	293	33.1	0.018^3^
Household has electricity* (%)	1004	85.9	295	81.7	0.080^3^
Index child birth weight (g)	970	2989 ± 426	188	2940 ± 463	0.144^2^
Index child length-for-age at 18 months (z-score)	914	-0.84 ± 1.0	130	-0.76 ± 1.0	0.370^2^

^1^ Values are mean ± standard deviation for continuous variables and percentage for dichotomous variables.

^2^ P-values for tests of difference in mean/percentage between follow-up and lost to follow-up samples from ordinary least squares regression.

^3^ P-values for tests of difference in mean/percentage between follow-up and lost to follow-up samples from logistic regression.

^4^ P-values for tests of difference in mean/percentage between follow-up and lost to follow-up samples from ordered logistic regression.

*Denotes baseline characteristics from the original randomized trial.

Tables 2 and 3 summarize the background characteristics of the LNS and non-LNS groups in the follow-up and lost to follow-up samples, respectively. In both samples, the groups were balanced among all baseline characteristics, and the significant differences in birth weight and LAZ at 18 months reflected differences in these outcomes that were due to the intervention.

**Table 2:** Characteristics of follow-up sample by intervention group.

Variable	LNS Group		Non-LNS Group		P-value
	N	Mean ± SD or %^1^	N	Mean ± SD or %^1^	
Index child age at investments data collection (y)	350	4.9 ± 0.6	657	4.9 ± 0.6	0.166^2^
Older sibling age at investments data collection (y)	148	8.8 ± 1.3	315	8.9 ± 1.3	0.804^2^
Younger sibling age at investments data collection (y)	122	1.6 ± 1.0	249	1.6 ± 1.0	0.674^2^
Index child male (%)	350	48.6	657	48.1	0.886^3^
Maternal parity at birth of index child (n)	350	2.3 ± 1.3	657	2.3 ± 1.3	0.695^4^
Maternal age* (y)	350	26.9 ± 5.5	657	26.8 ± 5.4	0.721^2^
Maternal education* (y)	349	7.4 ± 3.8	655	7.4 ± 3.5	0.728^2^
Maternal height (m) *	349	1.59 ± 0.05	656	1.59 ± 0.06	0.474^2^
Head of household female* (%)	349	23.2	653	27.6	0.135^3^
Household has electricity* (%)	349	84.2	655	86.7	0.284^3^
Index child birth weight (g)	335	3034 ± 409	635	2967 ± 433	0.019^2^
Index child length-for-age at 18 months (z-score)	318	-0.72 ± 0.99	596	-0.91 ± 1.0	0.008^2^

^1^ Values are mean ± standard deviation for continuous variables and percentage for dichotomous variables.

^2^ P-values for tests of difference in mean/percentage between follow-up and lost to follow-up samples from ordinary least squares regression.

^3^ P-values for tests of difference in mean/percentage between follow-up and lost to follow-up samples from logistic regression.

^4^ P-values for tests of difference in mean/percentage between follow-up and lost to follow-up samples from ordered logistic regression.

*Denotes baseline characteristics from the original randomized trial.

**Table 3:** Characteristics of lost to follow-up sample by intervention group.

Variable	LNS Group		Non-LNS Group		P-value
	N	Mean ± SD or %^1^	N	Mean ± SD or %^1^	
Index child male (%)	63	54.0	178	52.8	0.874^2^
Maternal parity at birth of index child (n)	90	2.2 ± 1.3	223	2.1 ± 1.2	0.434^3^
Maternal age* (y)	90	27.0 ± 6.2	223	26.2 ± 5.7	0.251^4^
Maternal education* (y)	83	7.0 ± 4.2	212	7.7 ± 4.0	0.160^4^
Maternal height* (m)	89	1.58 ± 0.05	222	1.59 ± 0.06	0.779^4^
Head of household female* (%)	83	39.8	210	30.5	0.129^2^
Household has electricity* (%)	83	83.1	212	81.1	0.690^2^
Index child birth weight (g)	44	3049 ± 401	144	2906 ± 474	0.073^4^
Index child length-for-age at 18 months (z-score)	30	-0.40 ± 1.17	100	-0.87 ± 1.0	0.031^4^

^1^ Values are mean ± standard deviation for continuous variables and percentage for dichotomous variables.

^2^ P-values for tests of difference in mean/percentage between follow-up and lost to follow-up samples from logistic regression.

^3^ P-values for tests of difference in mean/percentage between follow-up and lost to follow-up samples from ordered logistic regression.

^4^ P-values for tests of difference in mean/percentage between follow-up and lost to follow-up samples from ordinary least squares regression.

*Denotes baseline characteristics from the original randomized trial.

### Effect of the intervention on investments in the index child

The estimated effects of the provision of SQ-LNS to mothers during pregnancy and the first six months postpartum and then to their children from 6–18 months of age on investments in index children are shown in Table 4. In both the LNS group and the non-LNS group, reported rates and levels of investments across most investment domains were relatively high. With one exception, noted below, there were no differences in investments in the index child between the LNS group and the non-LNS group. These results did not change in any meaningful way when comparing across the three original intervention groups (S2 Table) or when adjusted for attrition using inverse probability weights (S3 Table).

**Table 4:** Investments in index children by intervention group.

Outcome	Outcome values	Percentage [n/N] or Mean ± SD [N]*		Marginal Effect of Treatment (95% CI)	P-value
		LNS Group	Non-LNS Group		
Birth spacing	No siblings within 48 mo	69.2 [216/312]	70.0 [432/617]	0.002 (-0.060, 0.064)	0.942^1^
	Next sibling 24–48 mo	26.0 [81/312]	24.0 [148/617]	-0.002 (-0.049, 0.046)	
	Next sibling < = 24 mo	4.8 [15/312]	6.0 [37/617]	-0.001 (-0.015, 0.014)	
First complementary food at 6 mo	Yes = 1; No = 0	74.9 [233/311]	67.9 [415/611]	0.062 (-0.0009, 0.124)	0.053^2^
Duration of breastfeeding	Number of months	20.0 ± 4.2 [312]	20.4 ± 4.1 [612]	-0.448 (-1.062, 0.166)	0.154^3^
Child covered by health insurance	Yes = 1; No = 0	75.9 [236/311]	74.1 [455/614]	0.018 (-0.041, 0.077)	0.549^4^
Mother has child’s health record	Yes = 1; No = 0	54.5 [170/312]	57.5 [355/617]	-0.031 (-0.100, 0.037)	0.370^5^
Bed net use the previous night	No bed net	60.0 [180/300]	62.4 [372/596]	-0.008 (-0.075, 0.058)	0.804^6^
	Untreated bed net	10.0 [30/300]	6.5 [39/596]	0.001 (-0.006, 0.008)	
	Treated bed net	30.0 [90/300]	31.0 [185/596]	0.008 (-0.052, 0.067)	
Age-appropriate schooling progression	Yes = 1; No = 0	90.3 [308/341]	90.5 [573/633]	0.005 (-0.022, 0.032)	0.718^7^
Attends a private school	Yes = 1; No = 0	83.9 [281/335]	86.5 [536/620]	-0.020 (-0.064, 0.025)	0.378^8^
Frequency of paternal financial support	Never	4.3 [11/259]	4.1 [22/542]	0.005 (-0.009, 0.019)	0.455^9^
	Sometimes	13.1 [34/259]	12.0 [65/542]	0.013 (-0.022, 0.049)	
	Often	6.6 [17/259]	5.4 [29/542]	0.005 (-0.008, 0.018)	
	Always	76.1 [197/259]	78.6 [426/542]	-0.023 (-0.085, 0.039)	

*For categorical outcomes, values are percentages [n in category/N in intervention group]. For count outcomes, values are means ± standard deviations [N in intervention group].

^1^P-value on treatment group indicator variable from ordered logistic regression adjusted for index child age, maternal parity at birth of index child, maternal height, female head of household, household electrification, maternal age, and maternal education.

^2^ P-value on treatment group indicator variable from logistic regression adjusted for index child age, maternal parity at birth of index child, maternal height, female head of household, household electrification, and maternal education.

^3^ P-value on treatment group indicator variable from Poisson regression adjusted for index child age, maternal parity at birth of index child, maternal height, female head of household, household electrification, maternal age, and maternal education.

^4^ P-value on treatment group indicator variable from logistic regression adjusted for index child age, maternal parity at birth of index child, maternal height, female head of household, household electrification, and maternal education.

^5^ P-value on treatment group indicator variable from logistic regression adjusted for index child age, index child gender, maternal parity at birth of index child, maternal height, female head of household, household electrification, and maternal education.

^6^P-value on treatment group indicator variable from ordered logistic regression adjusted for index child age, maternal parity at birth of index child, maternal height, female head of household, and household electrification.

^7^P-value on treatment group indicator variable from logistic regression adjusted for index child age, maternal parity at birth of index child, maternal height, female head of household, household electrification, and maternal age.

^8^P-value on treatment group indicator variable from logistic regression adjusted for index child age, maternal parity at birth of index child, maternal height, female head of household, household electrification, and maternal education.

^9^ P-value on treatment group indicator variable from ordered logistic regression adjusted for index child age, maternal parity at birth of index child, maternal height, female head of household, household electrification, and maternal education.

In the family planning domain, almost 70% of mothers in both groups reported that the index child had no younger siblings born within 48 months of the index child. Approximately a quarter of women reported that the index child had a younger sibling born between 2 and 4 years after the index child, and just over 5% of mothers reported the index child’s next youngest living sibling was born less than 2 years after the index child.

In the breastfeeding domain, 74.9% and 67.9% of mothers in the LNS and non-LNS groups, respectively, reported that complementary foods were first introduced when the index child was six months of age in accordance with the World Health Organization’s recommendation. This difference between groups was marginally statistically significant; mothers in the LNS group were approximately 6% more likely (p = 0.053) than mothers in the non-LNS group to report introducing complementary foods at six months of age. When comparing across the three intervention groups, however, this difference was not statistically significant (S2 Table). The intervention provided SQ-LNS to index children in the LNS group starting around six months of age, and this may have made it more likely that children in the LNS group received complementary foods at the recommended age. However, on average, mothers reported introducing complementary foods at 5.2 months of age in the LNS group and at 4.9 months of age in the non-LNS group, so while possible, it is not likely that any difference in the timing of the introduction of complementary foods was driven solely by this feature of the intervention. In terms of duration of breastfeeding, mothers reported that they breastfed the index child for approximately 20 months, on average, before completely weaning, and this investment did not differ between intervention groups.

In the health domain, approximately three-quarters of all index children were reportedly covered by the National Health Insurance Scheme, and slightly over half of the mothers in both groups were able to produce the index child’s health record. A majority of index children in both groups (60.0% in the LNS group and 62.4% in the non-LNS group) did not sleep under a bed net the night before the mother was interviewed.

Over 90% of index children in both groups were making age-appropriate progression in school at the time of the interview, and over 80% were reported to be attending a private school. Finally, approximately three-quarters of respondents in both groups reported that the index child’s father always provided financial support to pay for items like food, clothing, and school fees for the index child.

**Fig 2:**
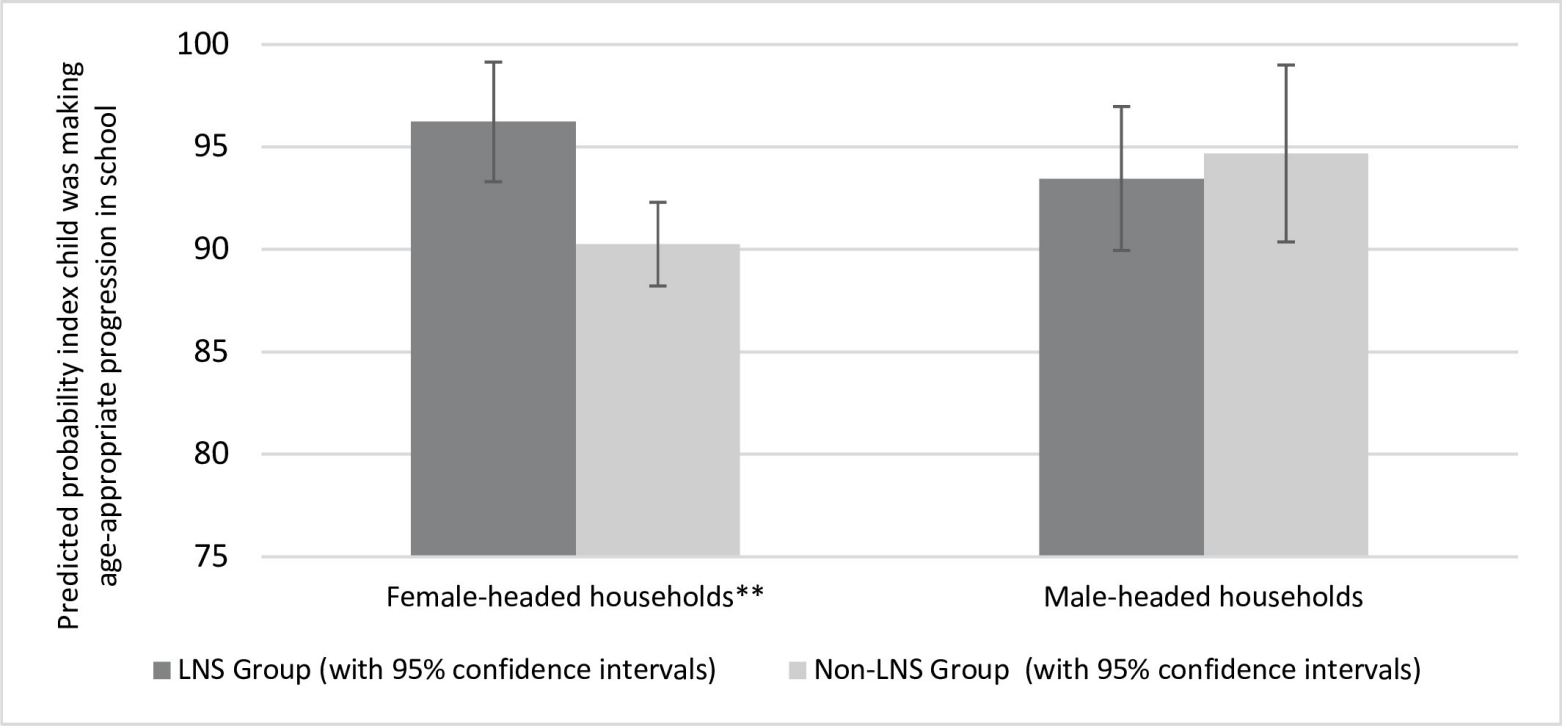
Heterogeneity, by the gender of the household head, in the effect of the intervention on the probability the index child was making age-appropriate progression in school. *** (p < .01), ** (p < .05), * (p < .1).

While the intervention generally did not affect parental investments in the index child, the effect of the intervention on whether the index child had completed at least an age-appropriate number of terms of schooling was modified by the gender of the household head (p-value for interaction = 0.042). Among female-headed households, index children in the LNS group were 6.0% more likely to have completed an age-appropriate number of terms of education than index children in the non-LNS group, while the difference was not significant in male-headed households (Fig 2). Other potential sources of heterogeneity examined, which were sex of the index child, maternal parity at the birth of the index child, maternal height, maternal age, maternal years of education, and whether the household had electricity, did not modify the effect of the intervention on any of the parental investments in the index child.

### Effect of the intervention on investment in siblings

Table 5 shows the estimated effects of the intervention on parental investments in the index child’s older siblings. Among older siblings, reported rates of health insurance coverage, maternal possession of the child’s heath record, and sleeping under a bed net were similar to reported rates for index children and did not differ between groups. In the education domain, there was no difference in the number of completed terms of education between groups, which was estimated using a negative binomial model given the ‘count’ nature of the outcome variable and the over-dispersion of its distribution. Likewise, there was no difference in the likelihood an older sibling was attending private school Again, these results did not change when comparing across the three intervention arms (S4 Table) or adjusting for attrition using inverse probability weights (S5 Table).

**Table 5:** Investments in older siblings by intervention group.

Outcome	Outcome values	Percentage [n/N] or Mean ± SD [N]*		Marginal Effect of Treatment (95% CI)	P-value
		LNS Group	Non-LNS Group		
Child covered by health insurance	Yes = 1; No = 0	75.2 [85/113]	67.5 [166/246]	0.066 (-0.041, 0.173)	0.243^1^
Mother has child’s health record	Yes = 1; No = 0	45.6 [52/114]	46.6 [117/251]	-0.035 (-0.155, 0.085)	0.566^2^
Bed net use the previous night	No bed net	56.7 [59/104]	59.5 [138/232]	-0.029 (-0.155, 0.098)	0.654^3^
	Untreated bed net	4.8 [5/104]	5.2 [12/232]	0.001 (-0.005, 0.008)	
	Treated bed net	38.5 [40/104]	35.3 [82/232]	0.027 (-0.093, 0.147)	
Completed terms of school	Number of terms	11.0 ± 3.8 [107]	11.0 ± 4.1 [247]	0.057 (-0.590, 0.703)	0.864^4^
Attends a private school	Yes = 1; No = 0	77.4 [82/106]	65.3 [160/245]	0.096 (-0.015 0.207)	0.107^5^

*For categorical outcomes, values are percentages [n in category/N in intervention group]. For count outcomes, values are means ± standard deviations [N in intervention group].

^1^P-value on treatment group indicator variable from logistic regression adjusted for age of sibling, age of index child, maternal parity at birth of index child, maternal height, female head of household, and household electrification. Standard errors clustered at household level.

^2^P-value on treatment group indicator variable from logistic regression adjusted for age of sibling, age of index child, maternal parity at birth of index child, maternal height, female head of household, household electrification, and maternal age. Standard errors clustered at household level.

^3^P-value on treatment group indicator variable from ordered logistic regression adjusted for age of sibling, age of index child, maternal parity at birth of index child, maternal height, female head of household, household electrification, sibling gender, and maternal education. Standard errors clustered at household level.

^4^P-value on treatment group indicator variable from negative binomial regression with exposure set to maximum terms possible and adjusted for age of sibling, age of index child, maternal parity at birth of index child, maternal height, female head of household, household electrification, sibling gender, maternal age, and maternal education. Standard errors clustered at household level.

^5^P-value on treatment group indicator variable from logistic regression adjusted for age of sibling, age of index child, maternal parity at birth of index child, maternal height, female head of household, household electrification, and maternal education. Standard errors clustered at household level.

**Fig 3:**
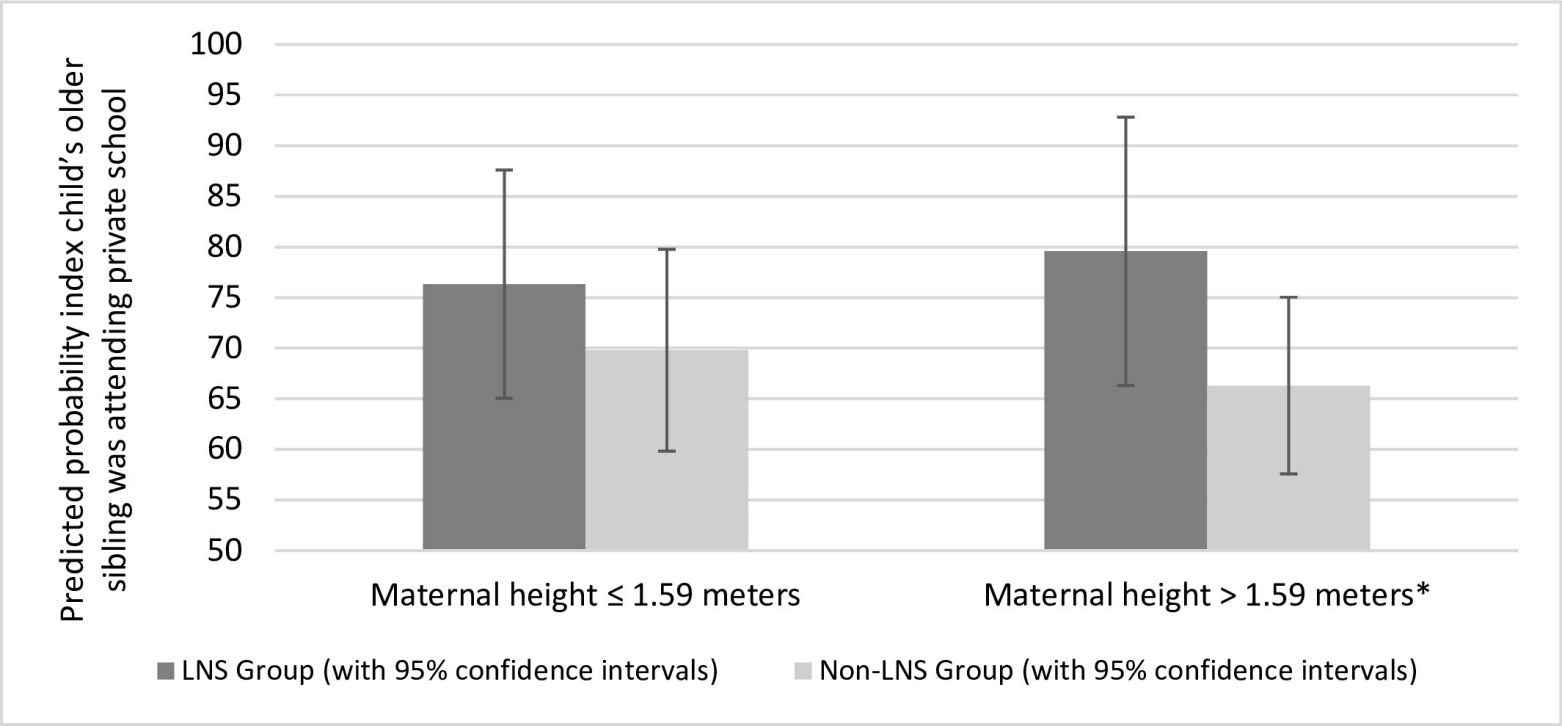
Heterogeneity, by maternal height, in the effect of the intervention on the probability the index child’s older sibling was attending private school. *** (p < .01), ** (p < .05), * (p < .1). None of the interaction effects for other potential effect modifiers (sex of the index child, sex of the older sibling, age of the older sibling, maternal parity at the birth of the index child, maternal age, maternal years of education, and whether the household had electricity) were statistically significant.

The effect of the intervention on private school attendance among older siblings was modified by maternal height (p-value for interaction = 0.048). Among older siblings with taller mothers (above the median maternal height), siblings of index children in the LNS group were 13.3% more likely to be attending a private school compared to the older siblings of children in the non-LNS group, while the difference was not statistically significant among older siblings of relatively shorter mothers (Fig 3).

**Fig 4:**
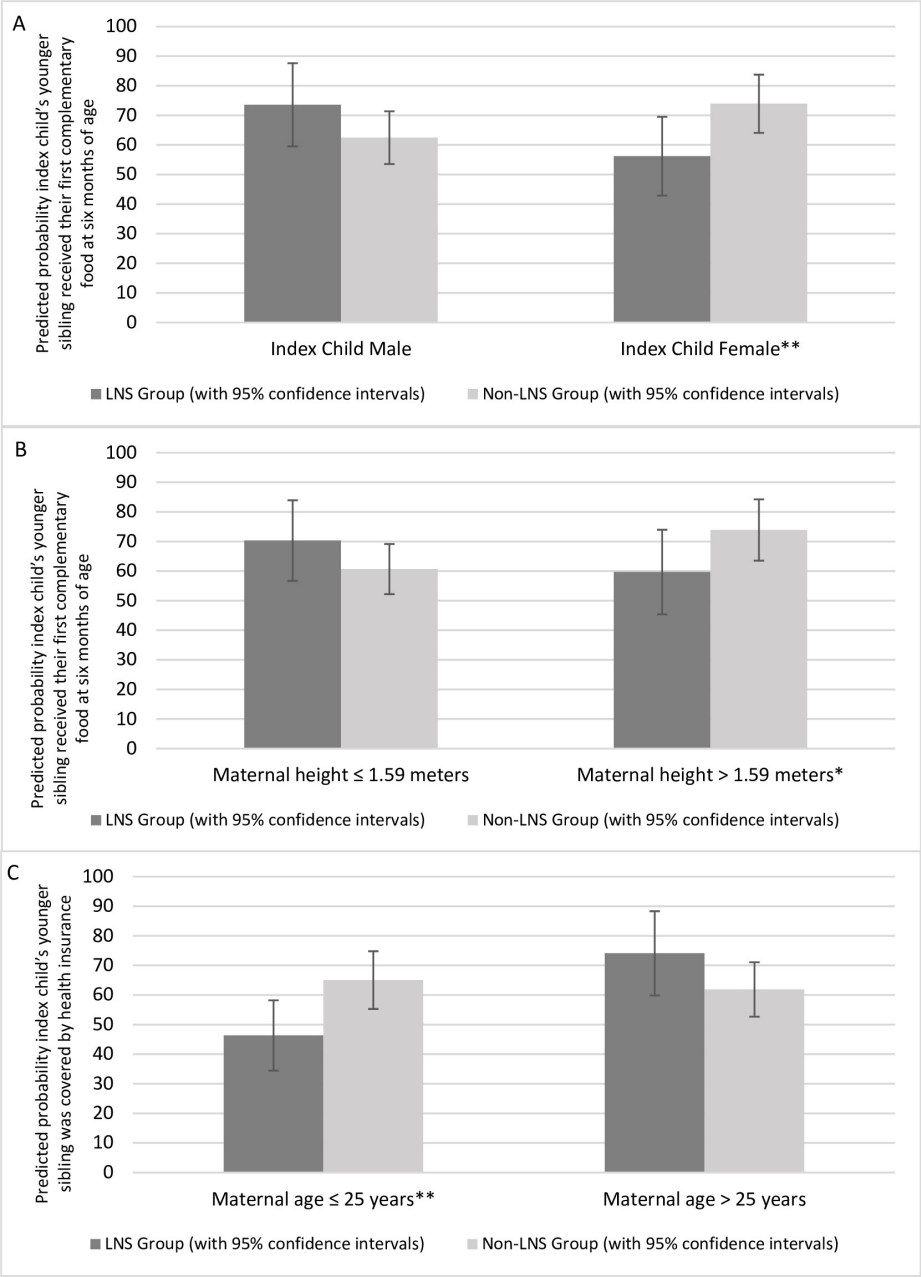
Heterogeneity in the effect of the intervention on investments in the index child’s younger sibling. (A) Heterogeneity, by index child gender, in the effect of the intervention on the probability the index child’s younger sibling received their first complementary food at six months of age. (B) Heterogeneity, by maternal height, in the effect of the intervention on the probability the index child’s younger sibling received their first complementary food at six months of age. (C) Heterogeneity, by maternal age, in the effect of the intervention on the probability the index child’s younger sibling was covered by health insurance. *** (p < .01), ** (p < .05), * (p < .1).

Among younger siblings, there were no differences in the timing of the introduction of complementary foods or in any of the health investments between the LNS and non-LNS groups (Table 6). These results did not change when comparing across the three original intervention groups (S6 Table) or when adjusting for attrition using inverse probability weights (S7 Table). There was, however, heterogeneity in several of the effects. First, younger siblings of female index children in the LNS group were 17.7% less likely to have received their first complementary food at six months of age compared to the non-LNS group, while the direction of the effect was positive, though not statistically significant, among younger siblings of male index children (p-value for interaction = 0.019) (Fig 4, Panel A). Maternal height also modified the effect of the intervention on the likelihood that the younger sibling received his/her first complementary food at six months of age (p-value for interaction = 0.037). The effect was negative and marginally significant for younger siblings with mothers above the median height but not significant among younger siblings with relatively shorter mothers (Fig 4, Panel B). Finally, maternal age modified the effect of the intervention on health insurance coverage for younger siblings (p-value for interaction = 0.018). Among younger mothers, the younger siblings of index children in the LNS-group were 18.7% less likely to be covered by health insurance than younger siblings of index children who did not receive SQ-LNS, while the difference was positive but not statistically significant among younger siblings with older mothers (Fig 4, Panel C). Other potential sources of heterogeneity (sex of the younger sibling, age of the younger sibling, maternal parity at the birth of the index child, maternal years of education, and whether the household had electricity) did not influence the effect of the intervention on parental investments in the younger sibling.

**Table 6:** Investments in younger siblings by intervention group.

Outcome	Outcome values	Percentage [n/N]*		Marginal Effect of Treatment (95% CI)	P-value
		LNS Group	Non-LNS Group		
First complementary food at 6 mo	Yes = 1; No = 0	63.8 [60/94]	67.4 [132/196]	-0.039 (-0.160, 0.082)	0.529^2^^,^^3^
Child delivered in a health facility	Yes = 1; No = 0	89.9 [107/119]	90.1 [219/243]	-0.014 (-0.079, 0.050)	0.660^1^
Child covered by health insurance	Yes = 1; No = 0	59.2 [71/120]	60.5 [147/243]	-0.031 (-0.148, 0.087)	0.608^4^
Mother has child’s health record	Yes = 1; No = 0	66.7 [80/120]	70.4 [171/243]	-0.037 (-0.142, 0.067)	0.476^5^
Bed net use the previous night	No bed net	59.2 [71/120]	54.8 [132/241]	0.067 (-0.039, 0.173)	0.222^6^
	Untreated bed net	10.0 [12/120]	5.8 [14/241]	-0.004 (-0.012, 0.004)	
	Treated bed net	30.8 [37/120]	39.4 [95/241]	-0.063 (-0.162, 0.036)	

*Values are percentages [n in category/N in intervention group].

^1^P-value on treatment group indicator variable from logistic regression adjusted for age of sibling, age of index child, maternal parity at birth of index child, maternal height, female head of household, and household electrification.

^2^P-value on treatment group indicator variable from logistic regression adjusted for age of sibling, age of index child, maternal parity at birth of index child, maternal height, female head of household, household electrification, and maternal age.

^3^Sample restricted to younger siblings who were six months of age or older on the date of enumeration.

^4^P-value on treatment group indicator variable from logistic regression adjusted for age of sibling, age of index child, maternal parity at birth of index child, maternal height, female head of household, household electrification, and maternal education.

^5^P-value on treatment group indicator variable from logistic regression adjusted for age of sibling, age of index child, maternal parity at birth of index child, maternal height, female head of household, household electrification, and sibling gender.

^6^P-value on treatment group indicator variable from ordered logistic regression adjusted for age of sibling, age of index child, maternal parity at birth of index child, maternal height, female head of household, and household electrification.

## Discussion

Undernutrition during the critical first 1,000 days can deprive children of the opportunity to achieve their full potential as healthy, productive adults [3]. As such, undernutrition experienced during this window can be thought of as a form of inequality that damages a child’s endowment, leaving him or her on unequal footing for the long-term. How parents respond to their child’s endowment has the potential to either exacerbate or reduce inequalities. Using data collected several years after a randomized trial that provided nutrient supplements to mothers during pregnancy and the first six months postpartum and then to their infants from 6–18 months of age, we explored whether parents’ investments in their children’s health and human capital changed as a result of the intervention. We also explored whether the intervention, which was provided to only one child in the household, affected investments in other children in the household.

Overall, we found that across the domains of family planning, breastfeeding, health, education, and paternal financial support, maternal and child supplementation with SQ-LNS did not change parental investments in either the children who received the supplements or their siblings. Among some subgroups, there was some evidence of higher investments in the education of index children and their older siblings in the group that received SQ-LNS, while some investments in breastfeeding and the health of younger siblings were lower in the group that received SQ-LNS. In some settings in Africa, children’s health and human capital outcomes have been shown to vary systematically based on a child’s sex and/or the sex composition of his/her siblings [31, 32]. In particular, a study in Ghana showed that children with all sisters (compared to all brothers) were predicted to have an approximately 25% higher height-for-age and up to a 40% higher weight-for-height. There is also evidence from the United States that the time a mother invested in her child reinforced the child’s early life endowment (as measured by birth-weight) among less-educated mothers (lower birth-weight children received less time), while time investments were compensating among more-educated mothers (lower birth-weight children received more time) [33]. However, considering these and other potential effect modifiers, we generally did not find evidence of heterogeneity in the impact of the intervention on parental investments in their children.

In the context of the intervention studied here, our results have several possible interpretations. The first possible interpretation is that, as has been found in several other LMIC contexts, given production technologies, constraints, and preferences, the intervention did not change parents’ optimal investment strategies. Another interpretation is that the effects of SQ-LNS on the index children’s endowments, namely increased birth size and attained length at 18 months of age, were too small for parents to perceive, or parents did perceive these differences but did not expect them to have any meaningful impact on the returns to investments in their children. Results of an assessment of parents’ perceptions of the intervention suggest that while most parents of children in the LNS group perceived that the supplements positively impacted the index child and expected continued positive impacts on the index child’s health and human capital into the future, parents in the group that received either iron-folic acid or multiple micronutrient capsules had similarly positive perceptions and expectations about the impacts of the supplements [34].

Our results are qualified by several limitations. First, the rate of attrition was higher in the non-LNS group compared to the LNS group, though the characteristics of the follow-up samples were well balanced between the LNS and non-LNS group in both the follow-up and lost to follow-up samples,. Additionally, while the rate of successful follow-up was relatively high, there were differences in the characteristics of the sample of children for whom investment data were collected at follow-up compared to those who were lost to follow-up. Nevertheless, we did control for baseline characteristic that differed between the samples, and adjusting for potential attrition using inverse probability weighting did not bring about any meaningful changes in the results. Second, our results are not adjusted for multiple hypothesis testing, so statistically significant effects should be interpreted with that caveat in mind. We defined five explicit outcome domains and limited the number of primary tests per domain, but the effect of the intervention on each outcome was also tested for heterogeneity (effect modification) across a number of baseline covariates. Although the outcomes and baseline covariates were pre-specified in an analysis plan, the threat of detecting a statistically significant effect by chance alone increases with each additional test [35]. A final limitation relates to the modest size of the effects of the intervention on birth weight and growth. While there is evidence that even relatively small shocks to a child’s endowment can have long-term effects [11], as noted above, we cannot determine whether our results reflect “neutral” parental investments (i.e., parents observed the shock to their child’s endowment but did not alter their investments) or whether the results were a consequence of the small effect sizes (i.e., parents did not observe the shock or the size of the shock was too small to be of consequence in decision-making).

Regardless of the reason for the limited effect of the intervention on parental investments in their children, our results are encouraging from a policy perspective. Theoretically and based on empirical evidence in the literature, it was possible that parents would have compensated for a positive shock to their child’s endowment by reducing investments in that child. If such reductions were likely to offset the long-term effects of the shock in a meaningful way, strategies to incentivize parents to increase investments in their children may have been necessary in order for the potential long-term benefits of a nutrition intervention like the provision of SQ-LNS to be fully realized. In this case, such additional incentives appear unnecessary. Our results suggest that parents in Ghana were generally making high levels of investments in their children’s health and human capital, including both the child who participated in the intervention and his/her siblings, and these investments did not change with the receipt of SQ-LNS.

## Supporting information

S1 Methods Information and nutrition messages. (DOCX)

S1 Table Nutrient composition of supplements. (DOCX)

S2 Table Investments in index children by original intervention group. (DOCX)

S3 Table Investments in index children by intervention group with inverse probability weighting. (DOCX)

S4 Table Investments in older siblings by original intervention group. (DOCX)

S5 Table Investments in older siblings by intervention group with inverse probability weighting. (DOCX)

S6 Table Investments in younger siblings by original intervention group. (DOCX)

S7 Table Investments in younger siblings by intervention group with inverse probability weights. (DOCX)

S1 Dataset Index_children. (DTA)

S2 Dataset Older_siblings. (DTA)

S3 Dataset Younger_siblings. (DTA)
