# Supplementary material for: The effects of a nutrient supplementation intervention in Ghana on parents’ investments in their children
Source: PLoS One. 2019 Mar 13;14(3):e0212178. doi: 10.1371/journal.pone.0212178 (PMC6415888; doi:10.1371/journal.pone.0212178)
Supplement: S1 Methods — (DOCX) [file pone.0212178.s001.docx]

**S1 Methods. Information and nutrition messages**

*Information Provided at Enrollment, with Basic Nutrition Message Repeated at 36 Weeks of Gestation*

To women who received capsules:

1. The capsules are all for you because women need more vitamins and minerals when they are pregnant.
2. You will have to take one capsule per day, every day in the week.
3. Do not share the capsules with others.
4. You will take the capsule with water in the morning after you have eaten. If you forget to take it in the morning, then take it in the afternoon after you have eaten. If you forget it in the morning and also in the afternoon, then take it in the evening after you have eaten. If you forget to take the capsule during the whole day, do not take two capsules the next day; it is always one capsule per day.
5. If you need to travel for a number of days, take the capsules with you so you can take them every day, whilst you are away.
6. Do not forget to eat meat, fish, eggs, fruits and vegetables whenever you can. You still need these foods even if you take the capsules we have given you.

To women who received Nkatepa (local name for SQ-LNS):

1. This Nkatepa supplement is all for you because women need more vitamins and minerals when they are pregnant.
2. You will have to eat one sachet per day, every day in the week.
3. Do not share the Nkatepa with others.
4. Mix the entire content of one sachet of Nkatepa with one ladle of food (any food you want) in the morning and eat. The one ladle of food is to make sure that you eat the amount of supplement you need for the day and not leave some of it mixed with food behind and let it go to waste. When that happens, it means you did not eat the amount of supplement your body needs for the day.
5. After you have eaten the one ladle of food mixed with the supplement, you can then go ahead and eat more of your food.
6. If you forget to eat the supplement in the morning, then eat it in the afternoon. If you forget it in the morning and also in the afternoon, then eat it in the evening.
7. If you forget the supplement the whole day, do not eat two sachets the next day; it is always one sachet per day.
8. If you need to travel for a number of days, take the Nkatepa with you so you can take them every day, whilst you are away.
9. Do not forget to eat meat, fish, eggs, fruits and vegetables whenever you can. You still need these foods even if you eat the Nkatepa we have given you.

*Information Provided at 6 Months Postpartum*

To women whose infants received no supplementation:

1. Breastfeed your baby as you did before.
2. Please do not forget to give your baby other things such as eggs, fruits and vegetables whenever you can. Your baby still needs these foods.

To women whose infants received Nkatepa (local name for SQ-LNS):

1. Breastfeed your baby as you did before.
2. This Nkpatepa supplement is for your baby because babies need special foods from 6 months of age; do not share it with others.
3. The baby will need to eat two (2) sachets per day, every day in the week. That is, you will give one sachet in the morning and another sachet in the afternoon or evening.
4. Each time you are giving the supplement to your baby, here is what you will do:
   - You will mix the entire content of the sachet with 2-3 tablespoons of already prepared food and feed it to the baby.
   - After the baby has eaten the 2-3 tablespoons mixed with the supplement, you can go ahead and give him/her more of the food. The 2-3 tablespoons is to make sure that the baby eats all of the supplement in the sachet and not leave some of it mixed with food behind and let it go to waste.
   - You can mix the Nkatepa with any food you are giving to the baby.
5. Do not cook food with the supplement; store the supplement at room temperature; you do not need to keep the supplement in the refrigerator.
6. If one day you did not give the supplement to your baby at all, or gave only one sachet instead of two sachets, do not give more than two sachets the next day; it is always two sachets per day.
7. In case you need to travel with the baby for a number of days, take the Nkatepa with you so you can give it to the baby every day, whilst you are away.
8. Do not forget to give your baby other things such as eggs, fruits and vegetables whenever you can. You baby still needs these foods even if you give him/her Nkatepa
