# Supplementary material for: The effects of a nutrient supplementation intervention in Ghana on parents’ investments in their children
Source: PLoS One. 2019 Mar 13;14(3):e0212178. doi: 10.1371/journal.pone.0212178 (PMC6415888; doi:10.1371/journal.pone.0212178)
Supplement: S1 Table — (DOCX) [file pone.0212178.s002.docx]

**S1 Table. Nutrient composition of supplements**

|  | Nutrient Content per Daily Ration | | | |
| --- | --- | --- | --- | --- |
|  | Infant SQ-LNS^1^ | Maternal SQ-LNS^1^ | Multiple Micronutrient Capsule | Iron-Folic Acid Capsule |
| Daily ration (g/day) | 20 | 20 |  |  |
| Total energy (kcal) | 118 | 118 |  |  |
| Protein (g) | 2.6 | 2.6 |  |  |
| Fat (g) | 9.6 | 10 |  |  |
| Linoleic acid (g) | 4.46 | 4.59 |  |  |
| α-Linolenic acid (g) | 0.58 | 0.59 |  |  |
| Vitamin A (µg RE) | 400 | 800 | 800 |  |
| Vitamin C (mg) | 30 | 100 | 100 |  |
| Vitamin B_1_ (mg) | 0.3 | 2.8 | 2.8 |  |
| Vitamin B_2_ (mg) | 0.4 | 2.8 | 2.8 |  |
| Niacin (mg) | 4 | 36 | 36 |  |
| Folic acid (µg) | 80 | 400 | 400 | 400 |
| Pantothenic acid (mg) | 1.8 | 7 | 7 |  |
| Vitamin B_6_ (mg) | 0.3 | 3.8 | 3.8 |  |
| Vitamin B_12_ (µg) | 0.5 | 5.2 | 5.2 |  |
| Vitamin D (IU) | 200 | 400 | 400 |  |
| Vitamin E (mg) | 6 | 20 | 20 |  |
| Vitamin K (µg) | 30 | 45 | 45 |  |
| Iron (mg) | 6 | 20 | 20 | 60 |
| Zinc (mg) | 8 | 30 | 30 |  |
| Copper (mg) | 0.34 | 4 | 4 |  |
| Calcium (mg) | 280 | 280 |  |  |
| Phosphorus (mg) | 190 | 190 |  |  |
| Potassium (mg) | 200 | 200 |  |  |
| Magnesium (mg) | 40 | 65 |  |  |
| Selenium (µg) | 20 | 130 | 130 |  |
| Iodine (µg) | 90 | 250 | 250 |  |
| Manganese (mg) | 1.2 | 2.6 | 2.6 |  |

^1^Nutrient contents for infant and maternal SQ-LNS include contributions from the food ingredients (vegetable oil, groundnut paste, sugar, and powdered milk) as well as from the multiple micronutrient premix.

Sources: [1, 2].

1. Adu-Afarwuah S, Lartey A, Okronipa H, Ashorn P, Peerson JM, Arimond M, et al. Small-Quantity, Lipid-Based Nutrient Supplements Provided to Women During Pregnancy and 6 mo Postpartum and to their Infants fom 6 mo of Age Increase the Mean Attained Length of 18-mo-old Children in Semi-Urban Ghana: A Randomized Controlled Trial. Am J Clin Nutr. 2016;104(3):797-808. doi: 10.3945/ajcn.116.134692

2. Adu-Afarwuah S, Lartey A, Okronipa H, Ashorn P, Zeilani M, Peerson JM, et al. Lipid-Based Nutrient Supplement Increases the Birth Size of Infants of Primiparous Women in Ghana. Am J Clin Nutr. 2015;101(4):835-46. doi: 10.3945/ajcn.114.091546
