# Supplementary material for: The effects of a nutrient supplementation intervention in Ghana on parents’ investments in their children
Source: PLoS One. 2019 Mar 13;14(3):e0212178. doi: 10.1371/journal.pone.0212178 (PMC6415888; doi:10.1371/journal.pone.0212178)
Supplement: S2 Table — (DOCX) [file pone.0212178.s003.docx]

**S2 Table. Investments in index children by original intervention group**

|  |  | Percentage [n/N] or Mean ± SD [N]* | | |  |
| --- | --- | --- | --- | --- | --- |
| Outcome | Outcome values | LNS Group | MMN Group | IFA Group | P-value |
| Birth spacing | No siblings within 48 mo | 69.2 [216/312] | 69.8 [220/315] | 70.2 [212/302] | 0.921^1^ |
|  | Next sibling 24-48 mo | 26.0 [81/312] | 24.1 [176/315] | 23.8 [72/302] |  |
|  | Next sibling <= 24 mo | 4.8 [15/312] | 6.0 [10/315] | 6.0 [18/302] |  |
| First complementary food at 6 mo | Yes = 1; No = 0 | 74.9 [233/311] | 67.9 [211/311] | 68.0 [204/300] | 0.152^2^ |
| Duration of breastfeeding | Number of months | 20.0 ± 4.2 [312] | 20.3 ± 4.0 [313] | 20.5 ± 4.1 [299] | 0.299^3^ |
| Child covered by health insurance | Yes = 1; No = 0 | 75.9 [236/311] | 78.3[244/313] | 69.9 [211/301] | 0.043^4^ |
| Mother has child’s health record | Yes = 1; No = 0 | 54.5 [170/312] | 54.6 [172/315] | 60.6 [183/302] | 0.229^5^ |
| Bed net use the previous night | No bed net | 60.0 [180/300] | 59.7 [181/304] | 65.2 [191/293] | 0.320^6^ |
|  | Untreated bed net | 10.0 [30/300] | 6.9 [21/303] | 6.1 [18/293] |  |
|  | Treated bed net | 30.0 [90/300] | 33.3 [101/303] | 28.7 [84/293] |  |
| Age-appropriate schooling progression | Yes = 1; No = 0 | 90.3 [308/341] | 91.8 [291/317] | 89.2 [282/316] | 0.497^7^ |
| Attends a private school | Yes = 1; No = 0 | 83.9 [281/335] | 85.3 [267/313] | 87.6 [269/307] | 0.541^8^ |
| Frequency of paternal financial support | Never | 4.3 [11/259] | 4.3 [12/277] | 3.8 [10/265] | 0.745^9^ |
|  | Sometimes | 13.1 [34/259] | 13.7 [38/277] | 10.2 [27/265] |  |
|  | Often | 6.6 [17/259] | 2.9 [8/277] | 7.9 [21/265] |  |
|  | Always | 76.1 [197/259] | 79.1 [219/277] | 78.1 [207/265] |  |

*For categorical outcomes, values are percentages [n in category/N in intervention group]. For count outcomes, values are means ± standard deviations [N in intervention group].

^1^P-value for Wald test of joint significance of intervention groups from ordered logistic regression adjusted for index child age, maternal parity at birth of index child, maternal height, female head of household, household electrification, maternal age, and maternal education.

^2^ P-value for Wald test of joint significance of intervention groups from logistic regression adjusted for index child age, maternal parity at birth of index child, maternal height, female head of household, household electrification, and maternal education.

^3^ P-value for Wald test of joint significance of intervention groups from Poisson regression adjusted for index child age, maternal parity at birth of index child, maternal height, female head of household, household electrification, maternal age, and maternal education.

^4^ P-value for Wald test of joint significance of intervention groups logistic regression adjusted for index child age, maternal parity at birth of index child, maternal height, female head of household, household electrification, and maternal education. Post-hoc pairwise group comparisons with Sidak’s adjustment for multiple comparisons: (1) MMN vs IFA group: estimated difference = 0.086; p = 0.05; (2) LNS vs IFA group: estimated difference = 0.06; p = 0.239; (3) LNS vs MMN group: estimated difference = -0.024; p = 0.85.

^5^ P-value for Wald test of joint significance of intervention groups from logistic regression adjusted for index child age, index child gender, maternal parity at birth of index child, maternal height, female head of household, household electrification, and maternal education.

^6^P-value for Wald test of joint significance of intervention groups from ordered logistic regression adjusted for index child age, maternal parity at birth of index child, maternal height, female head of household, and household electrification.

^7^P-value for Wald test of joint significance of intervention groups from logistic regression adjusted for index child age, maternal parity at birth of index child, maternal height, female head of household, household electrification, and maternal age.

^8^P-value for Wald test of joint significance of intervention groups from logistic regression adjusted for index child age, maternal parity at birth of index child, maternal height, female head of household, household electrification, and maternal education.

^9^ P-value for Wald test of joint significance of intervention groups from ordered logistic regression adjusted for index child age, maternal parity at birth of index child, maternal height, female head of household, household electrification, and maternal education.
