# Supplementary material for: The effects of a nutrient supplementation intervention in Ghana on parents’ investments in their children
Source: PLoS One. 2019 Mar 13;14(3):e0212178. doi: 10.1371/journal.pone.0212178 (PMC6415888; doi:10.1371/journal.pone.0212178)
Supplement: S3 Table — (DOCX) [file pone.0212178.s004.docx]

**S3 Table. Investments in index children by intervention group with inverse probability weighting**

|  |  | Percentage [n/N] or Mean ± SD [N]* | |  |  |
| --- | --- | --- | --- | --- | --- |
| Outcome | Outcome values | LNS Group | Non-LNS Group | Marginal Effect of Treatment (95% CI) | P-value |
| Birth spacing | No siblings within 48 mo | 69.1 [215/312] | 69.8 [430/617] | 0.004 (-0.057, 0.066) | 0.886^1^ |
|  | Next sibling 24-48 mo | 26.0 [81/312] | 24.2 [148/617] | -0.003 (-0.050, 0.043) |  |
|  | Next sibling <= 24 mo | 4.8 [15/312] | 6.1 [38/617] | -0.001 (-0.016, 0.013) |  |
| First complementary food at 6 mo | Yes = 1; No = 0 | 75.0 [233/311] | 68.0 [415/611] | 0.062 (0.001, 0.124) | 0.052^2^ |
| Duration of breastfeeding | Number of months | 20.0 ± 4.2 [312] | 20.4 ± 4.1 [612] | -0.457 (-1.022, 0.108) | 0.114^3^ |
| Child covered by health insurance | Yes = 1; No = 0 | 75.9 [236/311] | 74.2 [455/614] | 0.018 (-0.041, 0.077) | 0.561^4^ |
| Mother has child’s health record | Yes = 1; No = 0 | 54.5 [170/312] | 57.2 [353/617] | -0.029 (-0.097, 0.040) | 0.415^5^ |
| Bed net use the previous night | No bed net | 60.0 [180/300] | 62.1 [370/596] | -0.005 (-0.072, 0.061) | 0.877^6^ |
|  | Untreated bed net | 9.9 [30/300] | 6.5 [39/596] | 0.001 (-0.006, 0.007) |  |
|  | Treated bed net | 30.1 [90/300] | 31.4 [187/596] | 0.005 (-0.055, 0.065) |  |
| Age-appropriate schooling progression | Yes = 1; No = 0 | 90.3 [308/341] | 90.6 [574/633] | 0.004 (-0.023, 0.032) | 0.765^7^ |
| Attends a private school | Yes = 1; No = 0 | 84.0 [281/335] | 86.54 [536/620] | -0.018 (-0.062, 0.025) | 0.403^8^ |
| Frequency of paternal financial support | Never | 4.1 [11/259] | 4.0 [22/542] | 0.005 (-0.009, 0.019) | 0.443^9^ |
|  | Sometimes | 12.9 [34/259] | 11.9 [65/542] | 0.014 (-0.022, 0.049) |  |
|  | Often | 6.4 [17/259] | 5.5 [30/542] | 0.005 (-0.008, 0.018) |  |
|  | Always | 76.6 [201/259] | 78.6 [431/542] | -0.024 (-0.086, 0.038) |  |

*For categorical outcomes, values are inverse probability weighted (IPW) percentages [n in category/N in intervention group]. For count outcomes, values are inverse probability weighted means ± standard deviations [N in intervention group].

^1^P-value on treatment group indicator variable from IPW ordered logistic regression adjusted for index child age, maternal parity at birth of index child, maternal height, female head of household, household electrification, maternal age, and maternal education.

^2^ P-value on treatment group indicator variable from IPW logistic regression adjusted for index child age, maternal parity at birth of index child, maternal height, female head of household, household electrification, and maternal education.

^3^ P-value on treatment group indicator variable from IPW Poisson regression adjusted for index child age, maternal parity at birth of index child, maternal height, female head of household, household electrification, maternal age, and maternal education.

^4^ P-value on treatment group indicator variable from IPW logistic regression adjusted for index child age, maternal parity at birth of index child, maternal height, female head of household, household electrification, and maternal education.

^5^ P-value on treatment group indicator variable from IPW logistic regression adjusted for index child age, index child gender, maternal parity at birth of index child, maternal height, female head of household, household electrification, and maternal education.

^6^P-value on treatment group indicator variable from IPW ordered logistic regression adjusted for index child age, maternal parity at birth of index child, maternal height, female head of household, and household electrification.

^7^P-value on treatment group indicator variable from IPW logistic regression adjusted for index child age, maternal parity at birth of index child, maternal height, female head of household, household electrification, and maternal age.

^8^P-value on treatment group indicator variable from IPW logistic regression adjusted for index child age, maternal parity at birth of index child, maternal height, female head of household, household electrification, and maternal education.

^9^ P-value on treatment group indicator variable from IPW ordered logistic regression adjusted for index child age, maternal parity at birth of index child, maternal height, female head of household, household electrification, and maternal education.
