# Supplementary material for: The effects of a nutrient supplementation intervention in Ghana on parents’ investments in their children
Source: PLoS One. 2019 Mar 13;14(3):e0212178. doi: 10.1371/journal.pone.0212178 (PMC6415888; doi:10.1371/journal.pone.0212178)
Supplement: S4 Table — (DOCX) [file pone.0212178.s005.docx]

**S4 Table. Investments in older siblings by original intervention group**

|  |  | Percentage [n/N] or Mean ± SD [N]* | | |  |
| --- | --- | --- | --- | --- | --- |
| Outcome | Outcome values | LNS Group | MMN Group | IFA Group | P-value |
| Child covered by health insurance | Yes = 1; No = 0 | 75.2 [85/113] | 71.2 [84/118] | 64.1 [82/128] | 0.278^1^ |
| Mother has child’s health record | Yes = 1; No = 0 | 45.6 [52/114] | 45.9 [56/122] | 47.3 [61/129] | 0.839^2^ |
| Bed net use the previous night | No bed net | 56.7 [59/104] | 55.0 [61/111] | 63.6 [77/121] | 0.217^3^ |
|  | Untreated bed net | 4.8 [5/104] | 3.6 [4/111] | 6.1 [8/121] |  |
|  | Treated bed net | 38.5 [40/104] | 41.4 [46/111] | 29.8 [36/121] |  |
| Completed terms of school | Number of terms | 11.0 ± 3.8 [107] | 11.1 ± 4.2 [119] | 11.0 ± 4.1 [128] | 0.713^4^ |
| Attends a private school | Yes = 1; No = 0 | 77.4 [82/106] | 60.7 [71/117] | 69.5 [89/128] | 0.139^5^ |

*For categorical outcomes, values are percentages [n in category/N in intervention group]. For count outcomes, values are means ± standard deviations [N in intervention group].

^1^P-value for Wald test of joint significance of intervention groups from logistic regression adjusted for adjusted for age of sibling, age of index child, maternal parity at birth of index child, maternal height, female head of household, and household electrification. Standard errors clustered at household level.

^2^P-value for Wald test of joint significance of intervention groups from logistic regression adjusted for age of sibling, age of index child, maternal parity at birth of index child, maternal height, female head of household, household electrification, and maternal age. Standard errors clustered at household level.

^3^P-value for Wald test of joint significance of intervention groups from ordered logistic regression adjusted for age of sibling, age of index child, maternal parity at birth of index child, maternal height, female head of household, household electrification, sibling gender, and maternal education. Standard errors clustered at household level.

^4^P-value for Wald test of joint significance of intervention groups from negative binomial regression with exposure set to maximum terms possible and adjusted for age of sibling, age of index child, maternal parity at birth of index child, maternal height, female head of household, household electrification, sibling gender, maternal age, and maternal education. Standard errors clustered at household level.

^5^P-value for Wald test of joint significance of intervention groups from logistic regression adjusted for age of sibling, age of index child, maternal parity at birth of index child, maternal height, female head of household, household electrification, and maternal education. Standard errors clustered at household level.
