# Supplementary material for: The effects of a nutrient supplementation intervention in Ghana on parents’ investments in their children
Source: PLoS One. 2019 Mar 13;14(3):e0212178. doi: 10.1371/journal.pone.0212178 (PMC6415888; doi:10.1371/journal.pone.0212178)
Supplement: S6 Table — (DOCX) [file pone.0212178.s007.docx]

**S6 Table. Investments in younger siblings by original intervention group**

|  |  | Percentage [n/N]* | | |  |
| --- | --- | --- | --- | --- | --- |
| Outcome | Outcome values | LNS Group | MMN Group | IFA Group | P-value |
| First complementary food at 6 mo | Yes = 1; No = 0 | 63.8 [60/94] | 68.3 [71/104] | 66.3 [61/92] | 0.820^2,3^ |
| Child delivered in a health facility | Yes = 1; No = 0 | 89.9 [107/119] | 90.7 [117/129] | 89.5 [102/114] | 0.748^1^ |
| Child covered by health insurance | Yes = 1; No = 0 | 59.2 [71/120] | 60.5 [78/129] | 60.5 [69/114] | 0.765^4^ |
| Mother has child’s health record | Yes = 1; No = 0 | 66.7 [80/120] | 69.8 [90/129] | 71.1 [81/114] | 0.754^5^ |
| Bed net use the previous night | No bed net | 59.2 [71/120] | 54.7[70/128] | 54.9 [62/113] | 0.473^6^ |
|  | Untreated bed net | 10.0 [12/120] | 4.7 [6/128] | 7.1 [8/113] |  |
|  | Treated bed net | 30.8 [37/120] | 40.6 [52/128] | 38.1 [43/113] |  |

*Values are percentages [n in category/N in intervention group].

^1^ P-value for Wald test of joint significance of intervention groups from logistic regression adjusted for age of sibling, age of index child, maternal parity at birth of index child, maternal height, female head of household, and household electrification.

^2^P-value for Wald test of joint significance of intervention groups from logistic regression adjusted for age of sibling, age of index child, maternal parity at birth of index child, maternal height, female head of household, household electrification, and maternal age.

^3^Sample restricted to younger siblings who were six months of age or older on the date of enumeration.

^4^P-value for Wald test of joint significance of intervention groups from logistic regression adjusted for age of sibling, age of index child, maternal parity at birth of index child, maternal height, female head of household, household electrification, and maternal education.

^5^P-value for Wald test of joint significance of intervention groups from logistic regression adjusted for age of sibling, age of index child, maternal parity at birth of index child, maternal height, female head of household, household electrification, and sibling gender.

^6^P-value for Wald test of joint significance of intervention groups from ordered logistic regression adjusted for age of sibling, age of index child, maternal parity at birth of index child, maternal height, female head of household, and household electrification.
