# Supplementary material for: The effects of a nutrient supplementation intervention in Ghana on parents’ investments in their children
Source: PLoS One. 2019 Mar 13;14(3):e0212178. doi: 10.1371/journal.pone.0212178 (PMC6415888; doi:10.1371/journal.pone.0212178)
Supplement: S7 Table — (DOCX) [file pone.0212178.s008.docx]

**S7 Table. Investments in younger siblings by intervention group with inverse probability weights**

|  |  | Percentage [n/N]* | |  |  |
| --- | --- | --- | --- | --- | --- |
| Outcome | Outcome values | LNS Group | Non-LNS Group | Marginal Effect of Treatment (95% CI) | P-value |
| First complementary food at 6 mo | Yes = 1; No = 0 | 63.8 [60/94] | 67.1 [131/196] | -0.036 (-0.158, 0.085) | 0.554^2,3^ |
| Child delivered in a health facility | Yes = 1; No = 0 | 90.0 [107/119] | 90.2 [219/243] | -0.014 (-0.079, 0.050) | 0.654^1^ |
| Child covered by health insurance | Yes = 1; No = 0 | 59.3 [71/120] | 60.9 [148/243] | -0.030 (-0.145, 0.085) | 0.607^4^ |
| Mother has child’s health record | Yes = 1; No = 0 | 66.6 [80/120] | 70.1[170/243] | -0.037 (-0.144, 0.070) | 0.493^5^ |
| Bed net use the previous night | No bed net | 59.2 [71/120] | 54.6 [131/241] | 0.070 (-0.035, 0.174) | 0.196^6^ |
|  | Untreated bed net | 9.9 [12/120] | 5.8 [14/241] | -0.004 (-0.012, 0.003) |  |
|  | Treated bed net | 30.9 [37/120] | 39.6 [95/241] | -0.065 (-0.163, 0.032) |  |

*Values are inverse probability weighted (IPW) percentages [n in category/N in intervention group].

^1^P-value on treatment group indicator variable from IPW logistic regression adjusted for age of sibling, age of index child, maternal parity at birth of index child, maternal height, female head of household, and household electrification.

^2^P-value on treatment group indicator variable from IPW logistic regression adjusted for age of sibling, age of index child, maternal parity at birth of index child, maternal height, female head of household, household electrification, and maternal age.

^3^Sample restricted to younger siblings who were six months of age or older on the date of enumeration.

^4^P-value on treatment group indicator variable from IPW logistic regression adjusted for age of sibling, age of index child, maternal parity at birth of index child, maternal height, female head of household, household electrification, and maternal education.

^5^P-value on treatment group indicator variable from IPW logistic regression adjusted for age of sibling, age of index child, maternal parity at birth of index child, maternal height, female head of household, household electrification, and sibling gender.

^6^P-value on treatment group indicator variable from IPW ordered logistic regression adjusted for age of sibling, age of index child, maternal parity at birth of index child, maternal height, female head of household, and household electrification.
